# Endoscopic ultrasound-guided gallbladder drainage using a forward-viewing echoendoscope for Roux-en-Y reconstruction

**DOI:** 10.1055/a-2410-3830

**Published:** 2024-09-25

**Authors:** Ono Michihiro, Oiwa Shutaro, Yokoyama Ryota, Saito Seiya, Uesugi Atsushi, Abe Tomoyuki, Maeda Masahiro

**Affiliations:** 1 Department of Pancreatobiliary Medicine, Steel Memorial Muroran Hospital, Japan; 2 Department of Gastroenterology, Steel Memorial Muroran Hospital, Japan

Endoscopic ultrasound-guided gallbladder drainage (EUS-GBD) is an effective treatment option for high-risk surgical cholecystitis. However, Roux-en-Y (RY) reconstruction can be challenging because of the increased difficulty in reaching the duodenum 1 . Here, we report a case in which EUS-GBD was safely performed, using a forward-viewing echoendoscope (FV-EUS), in a patient with acute cholecystitis following RY reconstruction.

A 92-year-old woman with a history of total gastrectomy and RY reconstruction was admitted to our hospital with acute cholecystitis ( Fig. 1 ). She had an age-adjusted Charlson comorbidity index score of 6 and underwent percutaneous transhepatic gallbladder drainage (PTGBD). Choledocholithiasis was confirmed ( Fig. 2 ); a double-balloon endoscopy-assisted endoscopic retrograde cholangiopancreatography was performed on day 8 ( Fig. 3 ). Owing to a cystic duct stone, cholecystitis did not improve after PTGBD tube clamping ( Fig. 4 ). Therefore, we performed EUS-GBD using an FV-EUS (TGF-UC260J; Olympus Medical Systems, Tokyo, Japan). After careful manipulation, the duodenum was reached within 9 min. Injection of contrast medium from the PTGBD tube allowed the identification of the gallbladder lumen, as visualized on ultrasound imaging, enabling puncture using a 19G needle (EZ-shot 3 plus; Olympus). A self-expandable metal stent (BONASTENT M-Intraductal; Medico’s Hirata Inc., Osaka, Japan) was introduced and deployed over a 0.035-inch stiff guidewire (RevoWave SeekMaster Hard; Piolax Medical Devices, Kanagawa, Japan) after dilation using an 8-mm balloon catheter (REN biliary dilation catheter; KANEKA, Osaka, Japan) ( Video 1 ). Eight days later, the patient was discharged.

**Fig. 1:**
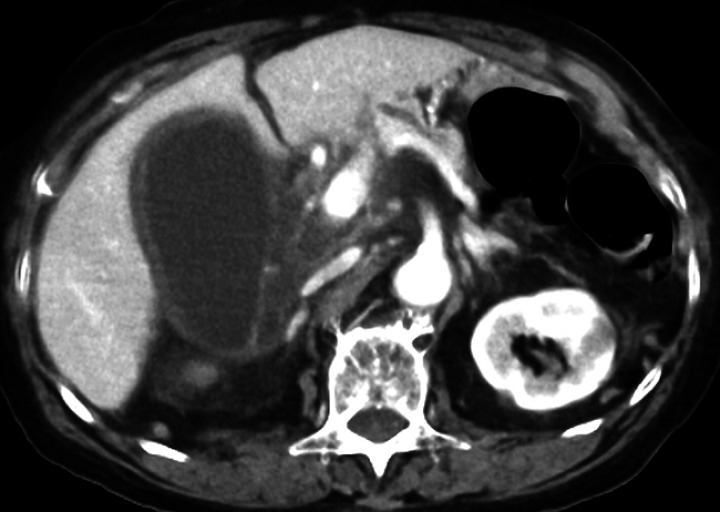
Computed tomography showing swelling of the gallbladder and thickening of the gallbladder wall.

**Fig. 2:**
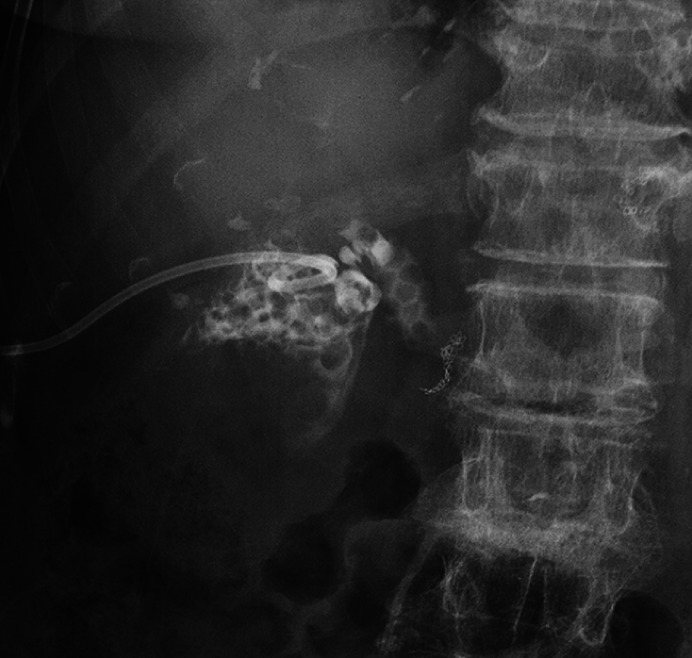
Contrast injection from the percutaneous transhepatic gallbladder drainage tube confirming the presence of choledocholithiasis.

**Fig. 3:**
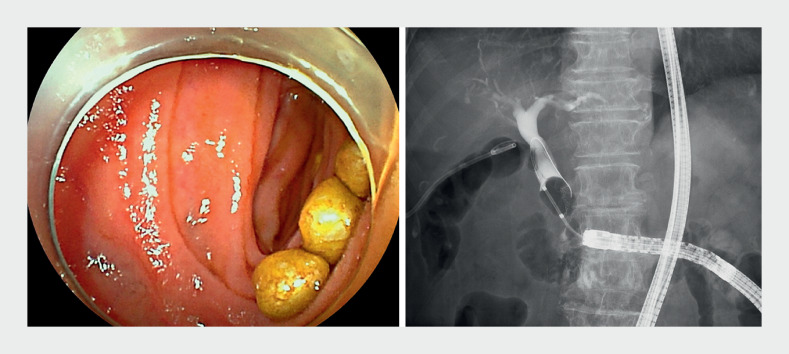
Contrast injection from the percutaneous transhepatic gallbladder drainage tube confirming the presence of choledocholithiasis.

**Fig. 4:**
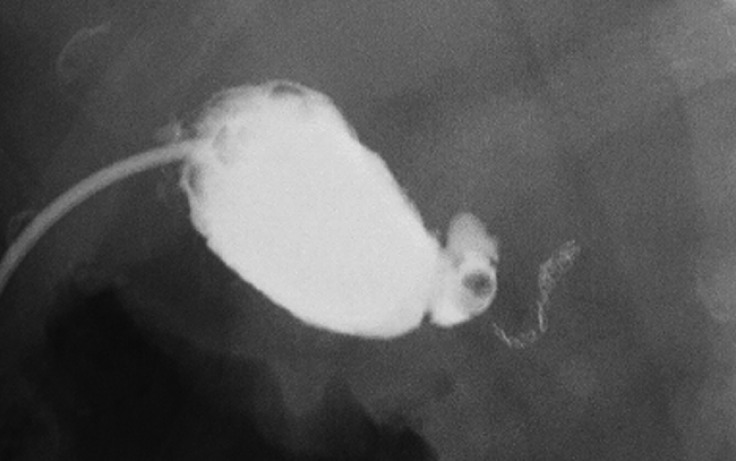
Repeat contrast injection through the percutaneous transhepatic gallbladder drainage tube revealing a stone impacted in the cystic duct.

A previously reported case of EUS-GBD following RY reconstruction with an oblique-viewing linear array echoendoscope highlighted the need for guidewire advancement 2 . The FV-EUS had greater flexibility and a shorter hard scope tip than that of the oblique-viewing echoendoscope ( Fig. 5 ), improving insertion capability and safety 3 and eliminating the need for guidewire advancement. To our knowledge, this is the first report of EUS-GBD using an FV-EUS for RY reconstruction.

**Fig. 5:**
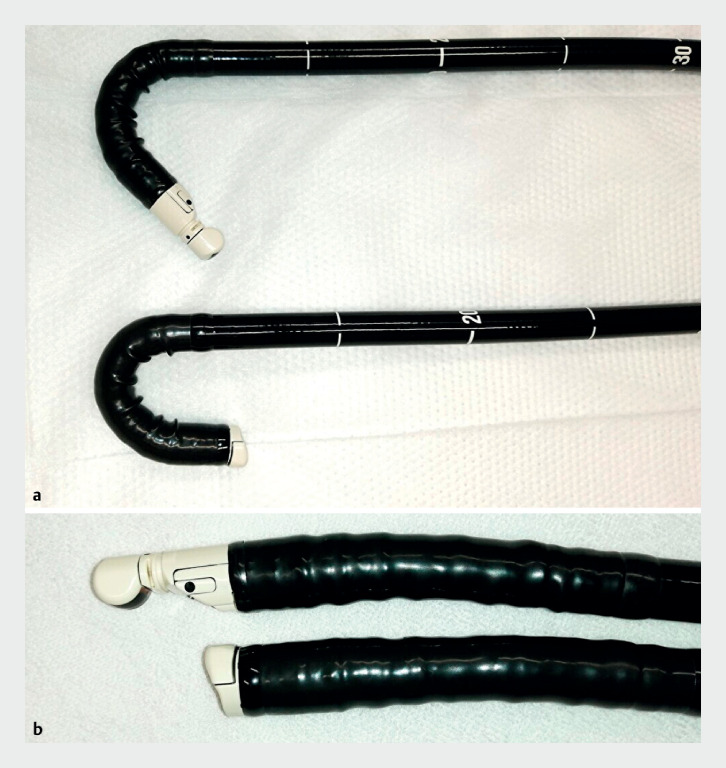
The forward-viewing echoendoscope has more flexibility and a shorter hard scope tip than that of the oblique-viewing echoendoscope.

Endoscopic ultrasound-guided gallbladder drainage using a forward-viewing echoendoscope in a patient with acute cholecystitis who underwent Roux-en-Y reconstruction: A case reportVideo 1

Endoscopy_UCTN_Code_TTT_1AS_2AH

## Notes

Endoscopy E-Videos https://eref.thieme.de/e-videos
*E-Videos* is an open access online section of the journal *Endoscopy* , reporting on interesting cases and new techniques in gastroenterological endoscopy. All papers include a high-quality video and are published with a Creative Commons CC-BY license. Endoscopy E-Videos qualify for HINARI discounts and waivers and eligibility is automatically checked during the submission process. We grant 100% waivers to articles whose corresponding authors are based in Group A countries and 50% waivers to those who are based in Group B countries as classified by Research4Life (see: https://www.research4life.org/access/eligibility/ ). This section has its own submission website at https://mc.manuscriptcentral.com/e-videos .
